# NK Cell Receptor/*H2*-D^k^–Dependent Host Resistance to Viral Infection Is Quantitatively Modulated by *H2* ^q^ Inhibitory Signals

**DOI:** 10.1371/journal.pgen.1001368

**Published:** 2011-04-21

**Authors:** Nassima Fodil-Cornu, J. Concepción Loredo-Osti, Silvia M. Vidal

**Affiliations:** 1 Department of Human Genetics and Department of Microbiology and Immunology, McGill University, Life Sciences Complex, Montreal, Canada; 2 McGill Centre for the Study of Host Resistance, McGill University, Montreal, Canada; 3 Department of Mathematics and Statistics, Memorial University of Newfoundland, St. Johns, Canada; The Jackson Laboratory, United States of America

## Abstract

The cytomegalovirus resistance locus *Cmv3* has been linked to an epistatic interaction between two loci: a Natural Killer (NK) cell receptor gene and the major histocompatibility complex class I (MHC-I) locus. To demonstrate the interaction between *Cmv3* and *H2^k^*, we generated double congenic mice between MA/My and BALB.K mice and an F_2_ cross between FVB/N (*H-2^q^*) and BALB.K (*H2^k^*) mice, two strains susceptible to mouse cytomegalovirus (MCMV). Only mice expressing *H2^k^* in conjunction with *Cmv3^MA/My^* or *Cmv3^FVB^* were resistant to MCMV infection. Subsequently, an F_3_ cross was carried out between transgenic FVB/*H2-D^k^* and MHC-I deficient mice in which only the progeny expressing *Cmv3^FVB^* and a single *H2-D^k^* class-I molecule completely controlled MCMV viral loads. This phenotype was shown to be NK cell–dependent and associated with subsequent NK cell proliferation. Finally, we demonstrated that a number of *H2^q^* alleles influence the expression level of *H2^q^* molecules, but not intrinsic functional properties of NK cells; viral loads, however, were quantitatively proportional to the number of *H2^q^* alleles. Our results support a model in which *H-2^q^* molecules convey Ly49-dependent inhibitory signals that interfere with the action of *H2*-D^k^ on NK cell activation against MCMV infection. Thus, the integration of activating and inhibitory signals emanating from various MHC-I/NK cell receptor interactions regulates NK cell–mediated control of viral load.

## Author Summary

Effective natural killer (NK) cell responses against virally infected cells are regulated by NK cell receptors that specifically recognize target cells. In the current study, we validated the specific interaction taking place between NK cell receptors and MHC class I molecules on the surface of infected cells, resulting in resistance to cytomegalovirus. Genetic dissection of this mechanism of interaction revealed that the NK cell response occurs exclusively through the triggering of the activating Ly49P receptor by the MHC class I *H2*-D^k^ molecule. We observed, in this context, that NK cells were incapable of clearing the virus when target cells also expressed MHC class I *H2^q^* molecules, which strongly and quantitatively inhibit NK cells. Our findings reveal that the interplay between inhibitory and activating NK cell receptors and their MHC class I ligands generate signals that shape the outcome of infection.

## Introduction

Natural killer (NK) cells play an important role in the innate immune response against tumors, MHC-mismatched bone-marrow grafts, and pathogens [1]–[2]. These cells also contribute to defense against parasites and intracellular bacteria, and they are critical for the control of a variety of viral infections [3]–[6]. NK cell actions are immediate and appear to be particularly important during the first few days of infection; they involve direct lysis of infected cells and production of proinflammatory cytokines [7]. NK cell activation is tightly regulated by output signals derived from the engagement of inhibitory and activating receptors by their respective ligands on potential targets [8]. Inhibitory human killer immunoglobulin–like receptors (KIR), mouse killer C-type lectin-like receptors family A (KLRA or Ly49), and NKG2A/CD94 receptors recognize major histocompatibility (MHC) class I molecules (*H2* in mice), thus controlling NK cell reactivity against “self.” As virally infected cells downregulate the expression of MHC class I molecules, the lack of inhibitory signals stimulates NK cells. This mechanism is described as the “missing self” hypothesis, whereby NK cells eliminate targets that lack normal levels of self-MHC class I molecules [9]. In addition, the interaction between inhibitory receptors and self MHC-I molecules is the basis of NK cell education (also termed licensing), leading to the maturation of functional NK cells in homeostatic conditions [10]–[17]. By contrast, several families of activating receptors, such as activating KLRA (also known as Ly49) receptors, KLRK1 (NKG2D) and the natural cytotoxicity receptor (NCR) NKP46 (NCR1) can induce NK cell activation through the recognition of viral ligands or stress-induced molecules [18]–[22]. Although it is clear that NK cell responses are modulated by a balance of opposing signals received from self- or nonself-specific ligands, the precise contribution of specific inhibitory and activating pathways to the resolution of infection remains to be fully understood.

The genetic dissection of host resistance or susceptibility to mouse cytomegalovirus (MCMV) has provided a fresh view of the precise role of activating NK cell receptors in the recognition of infected cells and host protection against the infection. Using informative crosses between various mouse strain combinations, several MCMV-resistance loci have been mapped to the NK cell gene complex (NKC) on mouse chromosome 6. The best characterized, *Cmv1* (also known as *Klra8*) and *Cmv3*, are defined by two different modes of inheritance, which seem to correlate with two different mechanisms of recognition. *Cmv1* is a single dominant locus whose resistance allele, described in C57BL/6 (B6) mice, encodes the Ly49H activating receptor. Ly49H recognizes MCMV-infected cells through a direct interaction with the viral product m157 [21]–[22]. Engagement of Ly49H by m157 elicits NK cell–mediated cytotoxicity, cytokine secretion, NK cell proliferation, and viral clearance [18], [23]–[24]. The *Cmv3* locus was detected in a cross between resistant MA/My and susceptible BALB/c mice. Expression of *Cmv3*-determined resistance accounted for a 100-fold decrease in splenic viral load, but it was only observed in mice carrying a specific combination of MA/My alleles at the NKC and MHC (*H2^k^*) loci. Functional candidate gene testing of Ly49 receptors isolated from MA/My mice showed that another DAP12-associated receptor, Ly49P, responded to MCMV-infected cells [25]. In this case, Ly49P functional recognition of target cells required surface expression of both the host *H2*-D^k^ molecule and the viral component *m04*/gp34 [26]. The role of the *H2^k^* haplotype in MCMV resistance was previously associated with greater survival following infection with lethal inoculum doses of MCMV compared to other *H2* haplotypes [27]. In addition, linkage analyses in a cross between resistant MA/My and susceptible C57L strains, as well as the generation of congenic C57L.M-*H2* ^k^ mice carrying the *H2* ^k^ allele from MA/My, confirmed a role for *H2*-D^k^-linked resistance to MCMV [28]–[29]. Nevertheless, the mechanism of resistance regulated by the interaction between NK receptors and MHC class I molecule is still unclear.

In MA/My mice, *Cmv3*-determined MCMV resistance served as a model for researchers and allowed them to propose the existence of a functional interaction between the activating Ly49P receptor on NK cells and MHC class I *H2*-D^k^ molecules. However, the role of a Ly49P-*m04*-*H2*-D^k^ stimulatory axis remained to be clarified. In the present study, we sought to replicate experimentally the statistical association between the NKC and the MHC-I locus in MCMV resistance, to determine the precise molecules involved in MCMV resistance *in vivo*, and to evaluate the impact of MHC-I inhibitory signals on the NK cell antiviral response.

## Results

### Interaction between NKC alleles and *H2^k^* locus is associated with resistance to MCMV

**Figure 1:**
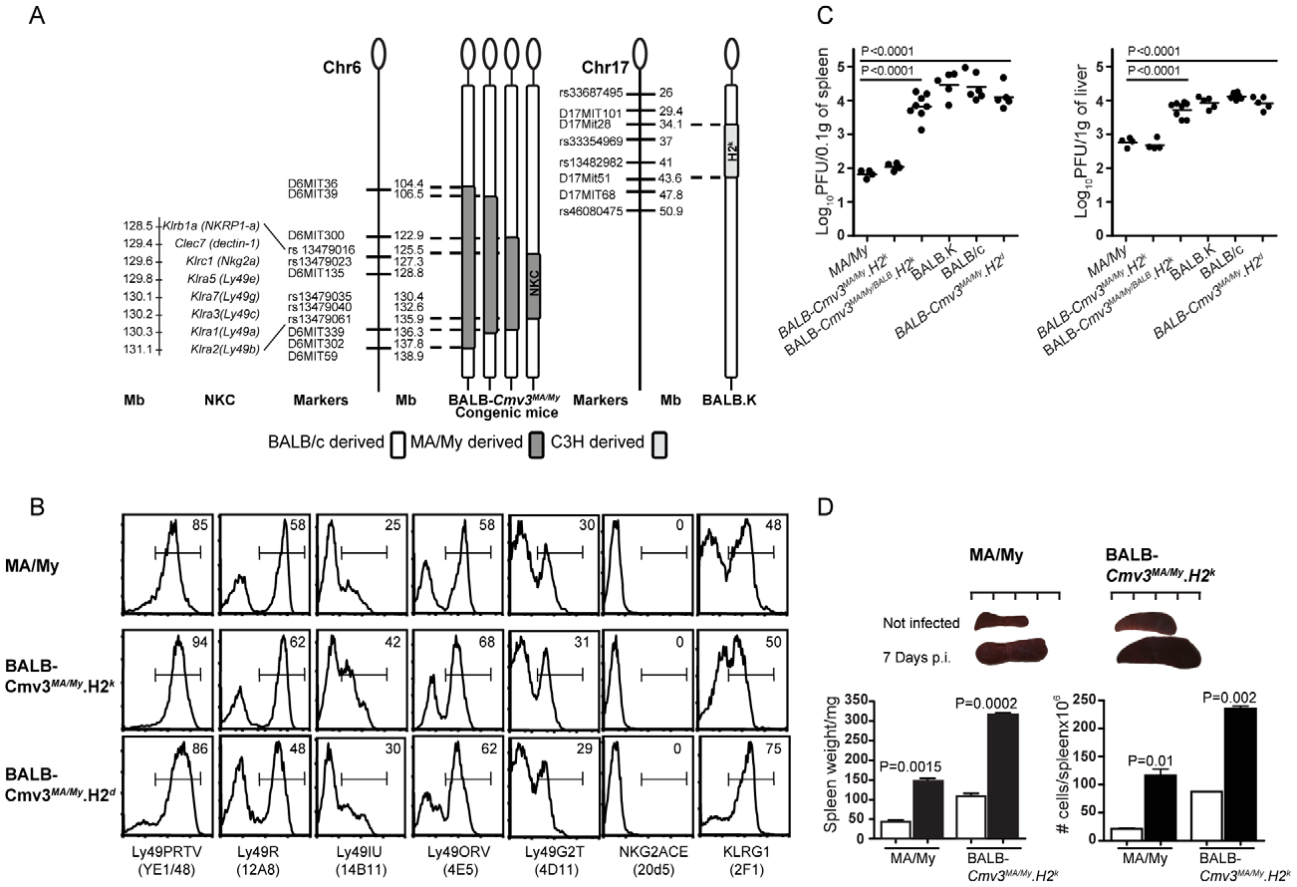
Generation, phenotype, and MCMV infection outcome of BALB mice congenic for the natural killer gene complex inherited from MA/My mice. (A) Left, physical map of chromosome 6 markers used to determine the size of the MA/My fragment introgressed into the BALB background. The four versions of chromosome 6 indicate the genotypes of sub-congenic strains produced during the generation of BALB.*Cmv3^MA^* ^/My^ congenic mice carrying a 10 Mb segment (between SNPs *rs13479016* and *rs13479061*) spanning the NKC region from parental MA/My mice. Right, physical map of chromosome 17 markers used to characterize the 9.4 Mb segment (between *D17Mit28* and *D17Mit51*) comprising the *H2* region of BALB.K mice (*H2^k^*). (B) NK cell receptor expression in MA/My parental mice and derived BALB-*Cmv3^MA/My^H2^d^* and BALB-*Cmv3^MA/My^H2^k^* congenic mice. The receptors (indicated on the bottom of the panel) were gated by FACS on NKp46^+^ splenic NK cells; the proportion of expression is indicated in each histogram. (C) Viral load in spleens (left) and livers (right) of mice of the indicated genotypes, as determined by plaque-forming assays 3 days p.i. (D) Spleen size (top) and weight and total cellularity (bottom) determined in MA/My and BALB-*Cmv3^MA/My^H2^k^* mice at 7 days p.i. White bar, uninfected mice; black bar, MCMV-infected mice. Data were analyzed using two-way ANOVA analysis and the two-tailed Student's test. Data are presented as mean ± SEM and *P* values of significant differences between groups are indicated. Results shown in panel B are representative of three experiments using 2–3 mice per group; results shown in panels C and D are representative of five independent experiments using 3–8 mice per group.

To validate the epistatic interaction between the NKC and *H2* detected by linkage analysis [25], we used a marker-assisted strategy to construct congenic mouse lines in which a chromosome 6 segment (*Cmv3*) from MA/My MCMV-resistant mice was independently introgressed into BALB/c (*H2^d^*) and BALB.K (*H2^k^*) susceptible backgrounds. Congenic BALB.K mice have been described previously [30]. The correlations between the current physical maps and the genomic region introgressed in the respective single and double congenic strains BALB-*Cmv3^MA/My^H2^d^* and BALB-*Cmv3^MA/My^H2^k^* are shown in Figure 1 and Table 1.

**Table 1:** Nomenclature, NKC/*H2* genotype, and MCMV titer of mouse strains used in this paper.

Standard nomenclature	Abbreviation used in this paper	NKC genotype	*H2* genotype	*H2*-D^k^ transgene	MCMV titer
BALB/c		BALB	*H2* ^d^		high
**MA/My**		**MA/My**	***H2*** **^k^**		**low**
C.C3-*H2^k^*	BALB.K	BALB	*H2* ^k^		high
**C.MA-Cmv3r.C3- ** ***H2^k^***	**BALB-** ***Cmv3^MA/My^H2^k^***	**MA/My**	***H2*** **^k^**	**−**	**low**
C.MA-Cmv3r.C-*H2^d^*	BALB-*Cmv3^MA/My^H2^d^*	MA/My	*H2* ^d^	−	high
FVB/N		FVB/N	*H2* ^q^		high
FVB-Tg(*H2*-D^k^) (funder #65864)	FVB-Tg(D^k^)^+^	FVB/N	*H2* ^q^	+	high
	FVB-Tg(D^k^)^−^	FVB/N	*H2* ^q^	−	high
**B6.129P-** ***H2*** **-D1^tm1Bpe^** ***H2*** **-K1^tm1Bpe^**	**B6.** ***H2*** **^0^**	**B6**	***H2*** **^0^ (MHC-I knock out)**	**−**	**low**
(FVB-Tg(*H2*-D^k^)1Sv x B6.129P-*H2*-D1^tm1Bpe^ *H2*-K1^tm1Bpe^)F_3_	***Cmv3^FVB^*** **/** ***H2*** **^0^/Tg(D^k^)^+^ (F_3_)**	**FVB/N**	***H2*** **^0^**	**+**	**low**
	*Cmv3^FVB^*/*H2* ^0^/Tg(D^k^)^−^ (F_3_)	FVB/N	*H2* ^0^	−	high
	*Cmv3^FVB^*/*H2* ^0/q^/Tg(D^k^)^+^ (F_3_)	FVB/N	*H2* ^0/q^	+	high
	*Cmv3^FVB^*/*H2* ^0/q^/Tg(D^k^)^−^ (F_3_)	FVB/N	*H2* ^0/q^	−	high
	*Cmv3^FVB^*/*H2* ^q^/Tg(D^k^)^+^ (F_3_)	FVB/N	*H2* ^q^	+	high

Text in Bold indicates mouse strains whose genotypes correlate with low viral titers.

To examine the effect of the genetic background on the expression of NKC-encoded receptors in the *Cmv3^MA^* ^/My^ region, we used a panel of antibodies with known antigen specificities (Figure S1) [31]. Though several anti-Ly49 antibodies are cross-reactive [31], we observed variations among the mouse strains in terms of frequency of Ly49 subpopulations. Compared to BALB-*Cmv3^MA/My^H2^d^* mice, BALB-*Cmv3^MA/My^H2^k^* animals had a significantly increased frequency of NK cells stained with the monoclonal antibodies 12A8 (against Ly49R; *P* = 0.02) and 14B11 (against Ly49I/U; *P* = 0.007) (Figure 1B and Figure S2). Notably, 14B11-stained NK cells were also significantly increased in BALB-*Cmv3^MA/My^H2^k^* mice compared to MA/My mice (*P* = 0.005). These results demonstrated the influence of *H2* alleles [32], as well as the influence of an additional non-*H2* mechanism, in the formation of the Ly49 repertoire. We also observed a highly significant increase in the frequency of NK cells labeled with 2F1 antibody (*P* = 0.004), which recognizes the maturation marker KLRG1, in BALB-*Cmv3^MA/My^H2^d^* mice compared to their *H2^k^* counterparts. Finally, we observed that NK cells from the three mouse strains that share the *Cmv3^MA^* ^/My^ allele lacked expression of NKG2A/C/E and CD94-associated receptors at the protein but not mRNA level (Figure 1B and Figure S3B). By contrast, there was a normal expression of these receptors in the FVB/N mouse strain, which carries an NKC haplotype similar to that of MA/My mice (Figure S3A and S3B).

To evaluate the effect of the transferred MA/My chromosome 6 (*Cmv3*) segment on the response of MCMV-susceptible BALB/c and BALB.K mice, we infected congenic and parental control mice by intraperitoneal (i.p.) inoculation of MCMV sublethal doses (Figure 1C). By day 3 post-infection (p.i), uncontrolled MCMV replication was observed in the spleen of susceptible BALB/c mice (log_10_ plaque-forming units [PFU] = 4.39±0.16), while MCMV-resistant MA/My mice had restricted viral replication, as shown by a >100-fold lower viral titer (log_10_ PFU = 1.81±0.05) than that seen in BALB/c mice. Single congenic BALB.K and BALB-*Cmv3^MA/My^H2^d^* mice had viral titers that were indistinguishable from those observed in BALB/c mice. More importantly, congenic mice with the BALB.K background, which carry only one copy of the MA/My NKC (BALB-NKC^BALB^ *Cmv3* ^MA/My^.*H2* ^k^), were as susceptible to MCMV as BALB/c, BALB.K, and BALB-*Cmv3^MA/My^H2^d^* mice. By contrast, double congenic BALB-*Cmv3^MA/My^H2^k^* mice had restricted virus growth to the same extent as resistant MA/My mice. Viral titers in the liver correlated with those observed in the spleens. Furthermore, by day 7 p.i., the virus was cleared from the spleen (unpublished data), which had undergone a massive increase in weight and cell number in MA/My and BALB-*Cmv3^MA/My^H2^k^* mice (Figure 1D). Collectively, these data demonstrate that the interaction between *Cmv3^MA^* ^/My^ and *H2^k^* confers MCMV resistance and is sufficient to explain the control of viral load observed in MA/My mice.

**Figure 2:**
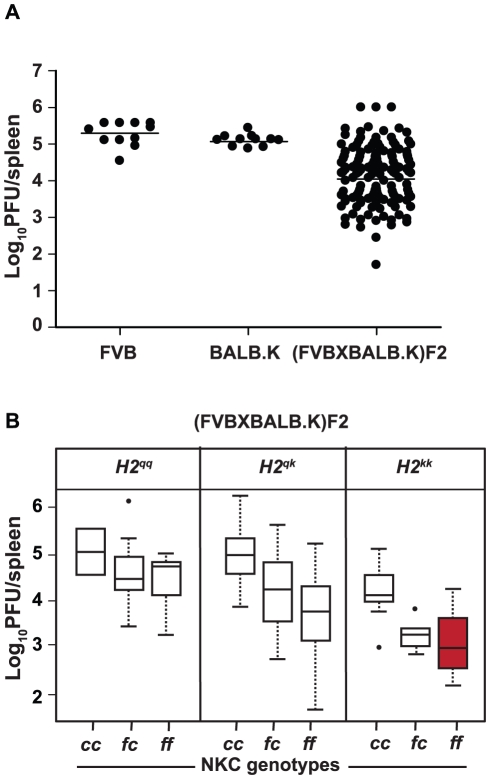
MCMV resistance in FVB/N×BALB.K F_2_ progeny dependent on specific combinations between the NKC and *H2*. (A) Genetic analysis of MCMV infection control in FVB/N×BALB.K F_2_ progeny. Mice (FVB/N and BALB.K, *n* = 11 per strain; FVB/N×BALB.K F_2_, *n* = 137) were infected with 5,000 PFU of MCMV; the spleen viral titers were determined by plaque assay at day 3 p.i. (B) Box plots show the combined effect of the NKC and *H2* loci derived from FVB/N (*H2^qq^* and *NKC^ff^*) or BALB.K (*H2^kk^* and *NKC^cc^*) parental mice on the viral loads in the spleens of the F_2_ progeny. The median and interquartile ranges are shown. Solid dots denote outliers. The red box corresponds to the combination of *NKC^ff^* and *H2^kk^* loci (*P*<6×10^−11^).

Because we did not have antibodies that specifically recognize Ly49P receptors and to examine a possible role of CD94 heterodimers, we attempted to confirm the results obtained in the congenic mice in a new cross between two strains that independently carried the *H2^k^* loci and the Ly49P gene at the NKC. We examined the segregation of MCMV viral load in the spleens of progeny mice from an F_2_ cross between the MCMV-susceptible strains FVB/N and BALB.K. Although both parental strains sustained a relatively high viral titer (5.2 log_10_ PFU), the 137 F_2_ progeny showed a continuous distribution ranging from 2 to 6 log_10_ PFU (Figure 2A). To evaluate the contribution of *H2* and NKC genes to MCMV resistance in this cross, F_2_ mice were genotyped and distributed according to their NKC (*Ly49e*) and *H2* (*IAA1*) genotypes. Mice homozygous for *H2^kk^* alleles from BALB.K and *NKC^ff^* alleles from FVB/N had the lowest viral load (Figure 2B). The model that best fitted this phenotype/genotype distribution in the analysis of variance had a joint logarithm of odds (LOD) score of 9 (*P*<10^−11^) and accounted for 29.6% of the phenotypic variation (Table 2). Thus, there was a highly significant association between NKC/*H2* interaction and control of MCMV infection in this second cross, indicating that *Cmv3* was also present in the FVB/N mouse strain and that its expression in the presence of *H2* ^k^ was necessary and sufficient for viral control. Furthermore, these data suggest that the same gene encodes *Cmv3* in both the MA/My and FVB/N NKC regions.

**Table 2:** ANOVA of MCMV load in spleens from (FVB/N×BALB.K) F_2_ mice.

Locus	*P* value	LOD	% Variance
Model	<6.0 E−11	9	29.6
*Ly49e* (NKC)	<4.5 E−5	7.2	20.7
*IAA1* (*H2*)	<6.4 E−5	3.2	8.9

### Transgenic expression of *H2-D^k^* has a modest effect on MCMV control in the presence of *H2^q^*

**Figure 3:**
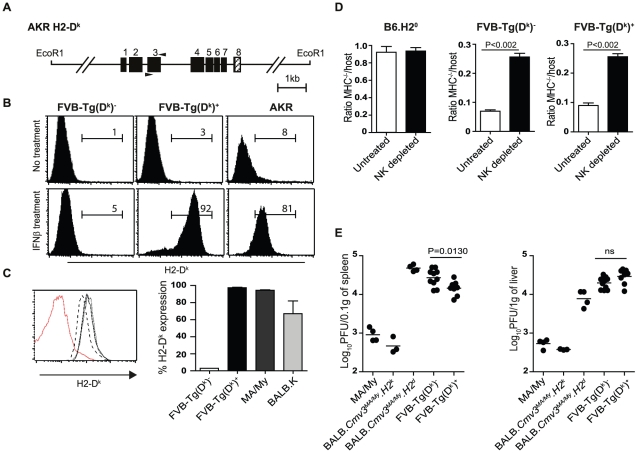
Functional characterization of *H2-D^k^* transgenic mice. (A) Schematic representation of the *H2-D^k^* gene within the 11.5 kb EcoRI genomic DNA fragment from AKR mice used to generate transgenic mice. (B) Expression of *H2-D^k^* on MEFs from FVB/N nontransgenic (FVB-Tg(D^k^)^−^, transgenic FVB-Tg(D^k^)^+^, and AKR (*H2^k^*) mice. MEFs from FVB-Tg(D^k^)^+^ and wild-type littermates were prepared from PCR-typed embryos, untreated or incubated overnight with 100 U/ml IFN-β prior to analysis of *H2*-D^k^ expression by FACS. (C) *H2*-D^k^ staining on lymphocytes from FVB-Tg(D^k^)^−^ (red peak), BALB.K (dashed peak), MA/My (black peak), and FVB-Tg(D^k^)^+^ (dotted peak) mice. Bar graph shows quantification of the percentage of *H2*-D^k^ expression from 2–3 mice per group. (D) Splenocytes from B6.*H2^0^* mice or NKC/*H2* histocompatible mice were inoculated into either untreated or asialo-GM1–treated NK cell–depleted hosts. Ratio values indicate the relative survival in the test population (CFSE^high^) compared to the histocompatible control population (CFSE^low^) at 18 hours after injection. Three mice per group were analyzed. Statistical significance between untreated and NK-depleted mice is shown. (E) Viral load in spleens (left) and livers (right) of mice of the indicated genotypes was determined by plaque-forming assays at day 3 p.i. Data were analyzed using two-way ANOVA analysis and the two-tailed Student's test. Data are presented as mean ± SEM and *P* values of significant results between groups are indicated. Results shown are representative of 2–3 independent experiments.

Previously, we showed that activation of Ly49P-bearing reporter cells requires the *H2*-D^k^ host molecule on MCMV-infected cells [25]–[26]. Therefore, to better delineate the role of *H2* in the host response against MCMV, we attempted *in vivo* rescue of the FVB/N susceptible phenotype by genetic transfer of an 11 kb *H2-D^k^* genomic fragment cloned from AKR mice (Figure 3A). We monitored for the presence of a diagnostic 300 bp fragment corresponding to exon 3 of the *H2*-D^k^ gene to identify transgenic FVB-Tg(D^k^)^+^ mice among the founder population (unpublished data). By surface staining of mouse embryo fibroblasts (MEF) from FVB-Tg(D^k^)^+^ mice, we observed the normal low levels of *H2*-D^k^ expression under regular conditions (Figure 3B). However, IFN-β treatment up-regulated expression of *H2*-D^k^ on MEF cells from either FVB-Tg(D^k^)^+^ mice or AKR mice (H2-D^k^ transgene donor mouse strain) to the same extent, indicating that the transgene promoter regulatory sequences were intact (Figure 3B). We also found that the level of expression of *H2*-D^k^ in splenocytes from the FVB/N transgenic mice was similar to the natural *H2*-D*^k^* expression in splenocytes from MA/My or BALB.K strains (Figure 3C). Finally, we investigated the expression of transgenic *H2*-D^k^ and endogenous *H2*-D*^q^* molecules in T and B cells isolated from the spleen and observed that the two MHC-I molecules were expressed in FVB-Tg(D^k^)^+^ mice at levels similar to those found in *H2^q^*- and *H2^k^*-bearing inbred mice (Figure S4). To test whether the *H2*-D^k^ transgene is capable of stimulating the Ly49P receptor, we assessed the activation of Ly49P-bearing reporter cells and found that these cells were equally activated by MCMV-infected MEF cells from BALB.K or from FVB-Tg(D^k^)^+^ (Figure S5A). Stimulation of Ly49P reporters was also observed upon challenge by MEFs infected with a mutant virus lacking m157 (the Ly49H ligand). However, Ly49P reporter cell stimulation was lost upon infection of MEF cells with a mutant virus lacking the *m04* gene, indicating that the transgenic H2-D^k^ molecule also requires *m04*/gp34 to stimulate Ly49P, as reported (Figure S5B) [26].

To establish the contribution of *H2*-D^k^ to the MCMV response, we first investigated a possible modulation of the Ly49 receptor repertoire. No significant differences were found between transgenic and non-transgenic mice in terms of the frequency of the various NK cell populations tested (Figure S6). Since *H2*-D*^k^* has the potential to influence licensing through its interactions with cognate Ly49 inhibitory receptors [16], we addressed the licensing status of wild-type and transgenic NK cells. To do this, we determined the ability of NK cells to mediate *in vivo* rejection of MHC class I deficient splenocytes isolated from B6.129P-*H2*-D1^tm1Bpe^ *H2*-K1^tm1Bpe^ (herein B6.*H2^0^*) mice using a quantitative CFSE-based method [33]. As previously described, B6.*H2^0^* mice tolerated grafted syngeneic splenocytes (Figure 3D). By contrast, both FVB-Tg(D^k^)^+^ and FVB-Tg(D^k^)^−^ mice rejected B6.*H2^0^* splenocytes with the same efficiency, suggesting that the presence of the *H2*-D*^k^* transgene does not alter the licensing status of NK cells (Figure 3D). Finally, we monitored early viral replication following MCMV infection in FVB-Tg(D*^k^*)*^+^* mice, along with single and double BALB-*Cmv3^MA/My^H2^d^* and BALB-*Cmv3^MA/My^H2^k^* congenic mice and parental MA/My mice. We observed that FVB-Tg(D*^k^*)*^+^* mice had a statistically significant, albeit modest, reduction in MCMV replication in the spleen (but not in the liver) compared to nontrangenic littermates; this reduction represented only a small fraction (1∶7) of the reduction observed in BALB-*Cmv3^MA/My^H2^k^* congenic mice (Figure 3E). Collectively, these data demonstrated that the *H2*-D*^k^* transgene was fully expressed and able to recognize and activate the Ly49P receptor *in vitro*; however, it only provided partial control of MCMV infection.

### Transgenic expression of *H2-D^k^* has a major effect on MCMV control in the absence of *H2^q^*

**Figure 4:**
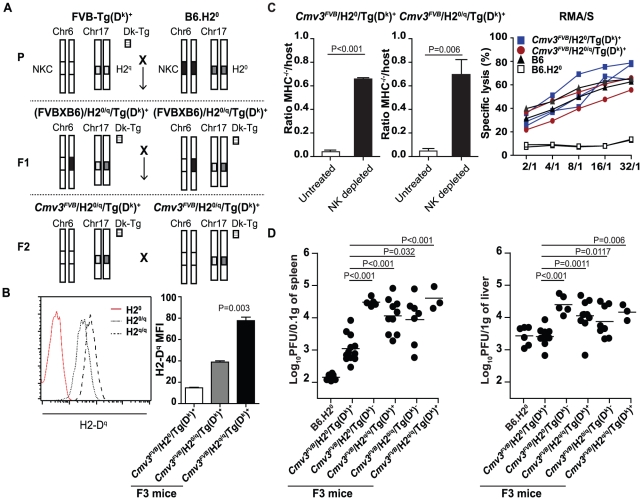
Functional characterization of F_3_ mice carrying the NKC from FVB/N and different assortment of *H2* molecules. (A) Breeding scheme for the generation of F_3_ mice carrying the NKC loci from FVB/N parental mice and various combinations of *H2* loci. The NKC, *H2*, and *H2*-D^k^ transgenic loci are represented by boxes, as indicated. The parental (P) FVB-Tg(D^k^)^+^ and B6.*H2^0^* strains were mated to generate the F_1_ generation. Subsequently, F_2_ mice carrying an homozygous FVB/N NKC locus and heterozygous for either the *H2* or the *H2-D^k^* transgene were kept and intercrossed to generate the F_3_ mice with different *H2* assortments (*H2^0^*: *H2-K^b^* ^−/−^ *D^b^* ^−/−^). (B) H2-D^q^ staining of lymphocytes from *Cmv3^FVB^/H2^0^*/Tg(D^k^)^+^ (*H2^0^* red peak), *Cmv3^FVB^/H2^0/q^*/Tg(D^k^)^+^ (*H2^0^* ^/q^, dot peak), and *Cmv3^FVB^/H2^q/q^*/Tg(D^k^)^+^ mice (*H2^q/q^*, dashed peak). Histograms on the right represent the quantification of the level of *H2*-D^q^ expression analyzed in three mice per group. (C) Rejection of B6 MHC class I–deficient cells *in vivo* by the indicated hosts was assessed as in Figure 3, and statistically significant differences are shown. IL-2–derived NK cells from the indicated mouse strains were co-cultured with CFSE-labeled RMA/S cells. Specific lysis at the indicated effector/target ratios was assessed by staining with 7-AAD and analyzed by FACS. Values represent the mean of 2–3 mice per group. (D) Viral loads in spleens (left) and livers (right) of parental and F_3_ mice of the indicated genotypes were determined by plaque assay at day 3 p.i. Results shown represent five pooled experiments. Data were analyzed using two-way ANOVA analysis and the two-tailed Student's test. Significant *P* values for differences between groups are indicated.

Classical MHC class I molecules are the prototype ligands for Ly49 receptors. Reporter cell assays and tetramer binding assays suggest that *H2*-D*^k^* molecules elicit both activating signals, through Ly49P, and inhibitory signals, through Ly49I and Ly49V [25], [31]. By contrast, *H2^q^*-encoded molecules can elicit strong inhibitory signals through Ly49I or Ly49C, but are inert to Ly49G2 [34], as well as to *Cmv3*-encoded activating receptors (Ly49P, Ly49R, and Ly49U) [25]. Therefore, we hypothesized that the poor MCMV infection control observed in FVB-Tg(D^k^)^+^ mice resulted from competition between the inhibitory and activating signals emanating from *H2^q^*- and *H2*-D*^k^*-encoded ligands, respectively. To test this hypothesis, we crossed FVB-Tg(D*^k^*)*^+^* transgenic mice with B6.*H2^0^* mice, which possess targeted deletions at the *H2-D* and *H2-K* genes. This cross produced F_3_ progeny mice homozygous for the FVB/N NKC (*Cmv3^FVB^*), but with a different assortment of MHC class I alleles. F_3_ mice were either (1) deficient in endogenous MHC class I alleles in the presence or absence of the *H2-D^k^* transgene (*H2^0^*/Tg(Dk)^+^ or *H2^0^*/Tg(Dk)^−^), (2) hemizygous for *H2^q^* in the presence or absence of the *H2-D^k^* transgene (*H2^0/q^*/Tg(Dk)^−^ or *H2^0/q^*/Tg(Dk)^+^), or (3) homozygous for *H2^q^* in the presence of the *H2-D^k^* transgene (*H2^q^*/Tg(Dk)^+^) (Figure 4A and Table 1). Again, we monitored whether the frequencies of various NK cell populations were affected by the genetic makeup of F_3_ mice and detected no major variations in the NK cell populations, the only exception being Ly49G^+^ NK cells, which were barely detectable in *Cmv3^FVB^*/*H2* ^0^/Tg(D^k^)^−^ mice (Figure S7). Similarly, the level of *H2*-D^k^ and *H2*-K^k^ expression on splenocytes was equivalent in transgenic F_3_ mice with different *H2* genotypes (Figure S7, right panel). By contrast, we noted that levels of H2-D^q^ expression on lymphocytes from homozygous *H2^q^*/Tg(D^k^)^+^ transgenic mice were double those of hemizygous *H2^0/q^*/Tg(D^k^)^+^ transgenic mice (*P* = 0.001) (Figure 4B and bar graph). Despite this variation in MHC class I expression levels, licensing of NK cells from H2-D^k^ transgenic mice carrying no (*0*), one (*0/q*), or two (*q/q*) *H2^q^* alleles was equivalent, as shown by their ability to reject CFSE-labeled splenocytes from B6.*H2^0^* mice [Figure 4C right]. These results were confirmed using explanted, IL-2-activated NK cells in cytotoxicity assays against MCH class I–deficient RMA/S target cells [Figure 4C left], demonstrating that NK cells from transgenic mice with different H2 assortments sense equally the loss of MHC class I expression on target cells and therefore are equally educated [16].

To determine the influence of the various MHC class I molecules on the NK cell immune response to MCMV infection, we examined F_3_ mice and parental controls at early time points, particularly on day 3 p.i., when receptor-specific NK cell responses are established [18]. On day 3, the post-infection viral titers in the spleens and livers of *Cmv3^FVB^*/*H2^0/q^*/Tg(D^k^)^+^ and *Cmv3^FVB^*/*H2* ^0^/Tg(D^k^)^−^ mice were indistinguishable, demonstrating that the presence of *H2^q^* dampens the effect of *Cmv3^FVB^*/*H2^k^* on the containment of virus replication (Figure 4D). By contrast, the presence of the transgene had a significant effect in the absence of endogenous class I molecules, as *Cmv3^FVB^*/*H2* ^0^/Tg(D^k^)^+^ mice had close to 30-fold lower viral titers compared to *Cmv3^FVB^*/*H2^0/q^*/Tg(D^k^)^−^ mice. In parallel, B6.*H2^0^* control mice, which express Ly49H, also cleared the virus load despite lacking MHC-I molecules (Figure 4D). To investigate the role of NK cells in limiting viral spread, we found that, as in MA/My mice, the control of virus load was abrogated in BALB-*Cmv3^MA/My^H2^k^* and *Cmv3^FVB^*/*H2* ^0^/Tg(D^k^)^+^ mice if treated with anti-asialo GM1 antibody prior to MCMV infection, demonstrating that the resistance phenotype is NK cell-dependent (Figure S8A). Indeed, we observed uncontrolled virus growth not only when MA/My mice were pretreated with anti-asialo GM1 and anti-NK1.1 antibodies, but also after they were pretreated with YE1/48 (anti-Ly49PRTV), 12A8 (anti-Ly49R), or 4D11 (anti-Ly49GT) antibodies, indicating an overlap in Ly49 receptor expression on NK cells (Figure S9) [25]. At day 6 p.i., a time characterized by robust proliferation of receptor-specific NK cell populations responding to the virus [18], we found that mice expressing *Cmv3* resistance (MA/My, BALB-*Cmv3^MA/My^H2^k^*, and *Cmv3^FVB^*/*H2* ^0^/Tg(D*^k^*)*^+^*) had cleared MCMV from the spleen (unpublished data). Furthermore, spleen cell numbers were increased 2–6-fold in these mice and BrdU uptake indicated a robust NK cell proliferation (Figure S8B). Together, our results indicate that expression of *H2*-D^k^ can rescue *Cmv3*-determined MCMV resistance in the absence of endogenous *H2* ^q^ molecules and that *Cmv3/H2-D^k^*-mediated resistance is associated with the expansion of NK cells in response to infection.

### Host *H2^q^* inhibitory signals quantitatively modulate MCMV resistance and restrict NK cell–specific proliferation upon MCMV challenge

**Figure 5:**
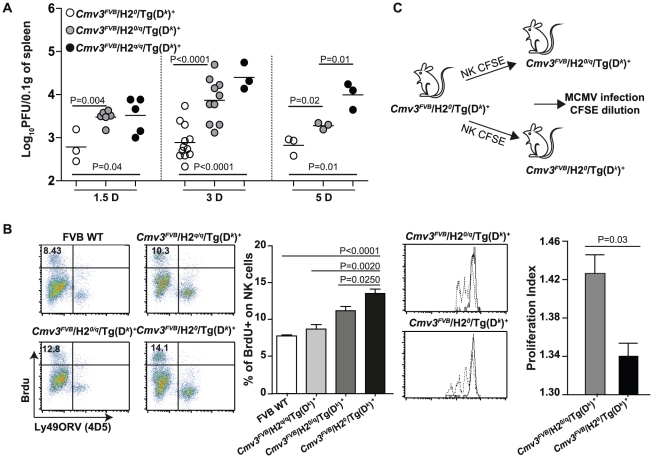
*H2^q^* expression interferes with NK cell antiviral responses. (A) MCMV viral load in the spleens of F_3_ transgenic mice at the indicated time-points was determined by plaque assay and *P* values of significant results between groups are indicated. (B) BrdU incorporation in NKp46-gated splenocytes stained with anti-BrdU and 4D5 antibodies. Splenoytes were isolated from mice of the indicated genotypes 5 days p.i. Graph bar represents the proportions of NK cells incorporating BrdU in total splenic leukocytes with standard deviations, using three mice per group. (C) Enriched NK cells from *Cmv3^FVB^/H2^0^*/Tg(D^k^)^+^ mice were labeled with CFSE then adoptively transferred into *Cmv3^FVB^/H2^0^*/Tg(D^k^)^+^ and *Cmv3^FVB^/H2^0/q^*/Tg(D^k^)^+^ recipients 24 hours before infection with MCMV for 5 days. Analysis of CFSE dilution in NK cells from the spleens of infected (dashed peaks) or uninfected (solid peaks) mice. NK cell proliferation index (number of divisions of CFSE-labeled NK cells) in *Cmv3^FVB^/H2^0^*/Tg(D^k^)^+^ and *Cmv3^FVB^/H2^0/q^*/Tg(D^k^)^+^ mice. Statistically significant differences between groups are indicated. Three mice per group were analyzed and results shown are representative of two experiments. Data were analyzed using two-way ANOVA analysis and the two-tailed Student's test. Significant *P* values for differences between groups are indicated.

To better define the role of *H2^q^* alleles, we compared the kinetics of viral replication in *Cmv3^FVB^*-Tg(D^k^)^+^ transgenic mice carrying no, one, or two *H2^q^* alleles. We observed that the number of *H2* alleles correlated with a quantitative increase in viral load, as early as 36 hours p.i.. On days 3 and 5 p.i., differences in viral containment among mice of the three genotypes were significant (Figure 5A).

**Figure 6:**
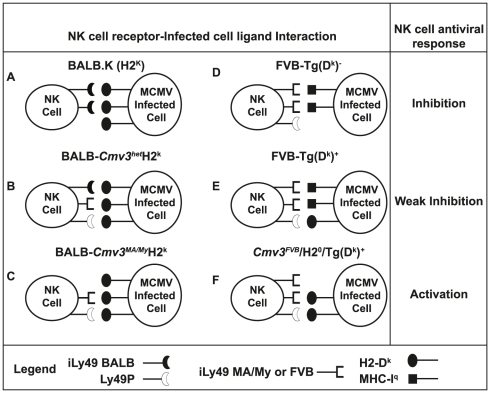
Model of *H2*-dependent, *Cmv3*-determined NK response against MCMV infection. The strength of Ly49 inhibitory signals and the presence of *H2*-D^k^-mediated activating signals modulate the NK cell response against virus infection. Our set of NKC congenic mice bore different assortments of Ly49 receptors, but carried an identical *H2^k^* resistance haplotype. (A) NK cells from BALB.K mice had a high frequency and strong binding of inhibitory Ly49 receptors, which rendered BALB.K mice most susceptible to MCMV infection. (B) NK cells from congenic BALB.*Cmv3^het^H2^k^* mice carried one copy of the activating *Ly49p* gene, which can activate the Ly49P/*H2*-D^k^/*m04* axis, allowing for intermediate viral loads in heterozygous mice. (C) NK cells from BALB.*Cmv3^MA/My^H2^k^* mice had the lowest frequency (and/or weakest binding) of inhibitory Ly49 receptors for *H2^k^* molecules and the highest frequency of activating Ly49P^+^ NK cells, resulting in strong control of MCMV infection. Our set of F_3_ mice carried different MHC-I components, but an identical *Cmv3*-resistance haplotype, encoding seven inhibitory and three activating Ly49 receptors, including Ly49P. (D) Engagement of inhibitory receptors in FVB-Tg(D*^k^*)*^−^* mice resulted in inhibition of the NK cell response against MCMV. (E) In FVB-Tg(D*^k^*)*^+^* mice, activating signals mediated by the engagement of Ly49P by *H2*-D^k^/*m04*, in the presence of inhibitory signals elicited by *H2^q^* molecules, provided a marginal enhancement of the NK cell response, and intermediate virus control. (F) In the absence of inhibitory *H2^q^* signals, *H2*-D^k^-dependent activation of NK cells was more efficient, which resulted in strong control of MCMV infection in *Cmv3^FVB^*/*H2^0^*/Tg(D*^k^*)*^+^* mice.

To investigate the effect of *H2^q^* molecules on NK cell specific responses against MCMV, we monitored BrdU incorporation on NK cells after MCMV infection in FVB/N WT and F_3_ mice carrying no, one, or two copies of *H2^q^* alleles. After 5 days p.i., NK cells were stained with the anti-Ly49ORV (4E5) monoclonal antibody, which stained around 50% of NK cells in these strains (Figure S6 and Figure S7), and with the anti-BrdU monoclonal antibody. In all mice, we observed that NK cells that incorporated BrdU were negative for the Ly49ORV antibody staining. Furthermore, the increase in BrdU incorporation was inversely proportional to the number of *H2^q^* alleles (Figure 5B). This result suggests that there is a dose-dependent inhibition of NK cell proliferation by *H2^q^* alleles in response to MCMV infection. Finally, to investigate whether host MHC-I molecules affect NK cell activity upon MCMV infection, we adoptively transferred CFSE-labeled NK cells enriched from *Cmv3^FVB^*/*H2* ^0^/Tg(D*^k^*)^+^ donor mice into *Cmv3^FVB^*/*H2* ^0^/Tg(D*^k^*)^+^ or *Cmv3^FVB^*/*H2* ^0/q^/Tg(D*^k^*)^+^ recipients (Figure 5C). After 5 days p.i., donor NK cells had undergone more rounds of division in the *Cmv3^FVB^*/*H2* ^0^/Tg(D*^k^*)^+^ recipients than the NK cells that were transferred into *Cmv3^FVB^*/*H2* ^0/q^/Tg(D*^k^*)^+^ mice; this indicated that *H2^q^* alleles limited NK cell proliferation induced by MCMV infection (Figure 6C bar graph). Thus, NK cells carrying *H2*-D^k^ as the sole MHC class I molecule were impaired in their ability to proliferate if the recipient mice carried *H2^q^* alleles. Collectively, these results suggest that expression of host *H2^q^* molecules dampens the capacity of NK cells to protect against MCMV.

## Discussion

In this study, we examined the combined contribution of the NKC and *H2* loci to the NK cell response against MCMV infection. We report that MCMV resistance was recapitulated in double-congenic mice and in an independent F_2_ cross that reconstituted the combination of *Cmv3* resistance alleles and *H2^k^*. Furthermore, we established that the *H2*-D^k^ molecule is essential to the resistance phenotype, because genetically susceptible mice bearing *Cmv3* were rendered resistant by acquisition of an *H2*-D^k^ transgene. However, efficient virus control was observed only in the absence of endogenous *H2^q^* molecules, whose inhibitory input quantitatively modulated virus control. Thus, MHC class I molecules play antagonistic roles in the NK response against viral infection.

### Combined effect of the NKC and MHC loci on NK cell antiviral responses

The role of the MHC has been studied using panels of congenic [27], sub-congenic, and transgenic mice or F_2_ crosses with the same NKC haplotype [28]–[29], [35]. To the best of our knowledge, the present study is the first to provide formal proof of the impact of both NKC and MHC haplotypes on NK cell antiviral activities *in vivo*. Our study, through the use of single- and double-congenic mice, minimized differences in non-NKC or non-MHC genes. Thus, we established that the joint action of specific alleles at these two regions accounted for most of the overall phenotypic differences between the MCMV-susceptible BALB/c and MCMV-resistant MA/My mouse strains. BALB-*Cmv3^MA/My^H2^k^* mice were indistinguishable from MA/My mice in terms of initial control of infection and late NK cell responses.

Although the MA/My NKC region in congenic mice encompasses more than just *Ly49* genes, our data indicates that the influence of MHC alleles on MCMV-resistance stems from the capacity of MHC class I molecules to serve as ligands for Ly49 receptors. In support of this hypothesis, the F_2_ cross between the MCMV-susceptible FVB/N (*H2^q^*) and BALB.K (*H2^k^*) mouse strains demonstrated that FVB/N mice carried a *Cmv3* resistance allele that was conditional to *H2^k^* and overridden by the *H2^q^* susceptibility allele. Within the *Cmv3* region, Ly49 receptors were responsive to MHC class I ligands. On the one hand, we noticed that none of the available anti-NKG2 or anti-CD94 antibodies recognized MA/My mouse NK cells, in contrast to NK cell recognition in FVB/N mice. Nevertheless, we observed that F_2_ mice of the combined *Cmv3^FVB^*/*H2^k^* genotype restrained viral replication to an extent similar to that seen in MA/My mice. On the other hand, our haplotype studies [25], [36] and new public data (http://phenome.jax.org) indicate that FVB/N and MA/My share the same *Ly49* gene repertoire, including Ly49P. Consequently, it seems that NK cell responsiveness during MCMV infection varies with different NKC-MHC combinations and is optimal only with a precise combination of Ly49 receptors inherited from MA/My (or FVB/N) mice and MHC class I *H2* ^k^ molecules.

### Role of the MHC class I molecule H2-D^k^

Our results confirmed that the *H2* effect was due to the MHC class 1 molecule *H2*-D^k^. Using an 11 kb genomic fragment containing a functional *H2-D^k^* gene, we achieved a phenotypic rescue, although the rescue was incomplete if combined with *H2^q^* alleles. The complete protective effect of *H2-D^k^* was restored in F_3_ mice lacking endogenous *H2^q^* molecules. Although *H2*-D^k^ also affects the adaptive immune response, early containment of viral replication, massive NK cell proliferation, and reversal of the resistance phenotype by depletion of NK cells in FVB-*H2^0^*-Tg(D^k^)^+^ clearly support a mechanism at the level of NK cells. Because of the presence of both inhibitory and activating Ly49 receptors, several nonexclusive scenarios could account for the precise mode of action of the combined MHC class I *H2-D^k^* and *Ly49* genotypes on the NK cell response against MCMV: (1)low threshold of NK cell activation through weak *H2*-D^k^/Ly49 inhibitory signals, (2) effective NK cell activation through *H2*-D^k^/Ly49 activating signals, and (3) interplay between *H2*-D^k^/Ly49 activating and inhibitory signals.

### Inhibitory signals

One possibility is that MHC class I/inhibitory Ly49 signals have a negative impact on the NK cell response to MCMV. In our study, mature NK cells in BALB.K mice (which are the most susceptible to MCMV infection) express three inhibitory receptors: Ly49A, Ly49C, and Ly49G2, which all bind to MHC-I *H2^k^* molecules [31], [34], [37]. Thus, the majority of NK cells from BALB.K mice should be inhibited by a receptor for a self-ligand. Indeed, we have recently shown that deletion of the *m04* gene renders BALB.K mice resistant to MCMV infection, as the protein it encodes abolishes NK cell activation via the “missing-self” recognition mechanism (Babic et al., 2010) [56]. The *m04*/gp34 protein escorts MHC class I molecules to the surface of infected cells, thus maintaining a level of surface MHC expression sufficient enough to trigger inhibitory NK cell receptors [38]. Thus, with only three (Ly49V, Ly49I, and Ly49G2) out of seven Ly49 inhibitory receptors able to recognize *H2*-D^k^ molecules, NK cells from BALB.*Cmv3^MA/My^ H2^k^* mice should be less susceptible to inhibition by *H2^k^* binding (Figure 6A–6C).

### Activating signals

The existence of an *H2*-D^k^–mediated activating axis to MCMV resistance is supported by the gain-of-function phenotype of FVB-*H2*-D^k^ transgenic mice, which presented itself despite their Ly49 repertoire that is virtually identical to that of their non-transgenic littermates (Figure S6A and S6B). Furthermore, the absence of NK cell triggering through inhibitory Ly49 receptors was not sufficient to allow efficient control of MCMV replication, as demonstrated by the F_3_ *Cmv3^FVB^* MHC class I–deficient mice. Most NK cells that develop in MHC class I–deficient hosts are unable to respond to MHC class I–deficient targets. However, a recent study demonstrated that, in the context of MCMV infection, NK cells eliminate virally infected cells in MHC class I–negative hosts, in addition to regaining the ability to eliminate MHC class I–deficient hematopoietic host cells [39]. This mechanism seems to be triggered by the inflammatory milieu induced by MCMV infection [39]. These observations suggest that the susceptibility of *Cmv3^FVB^* MHC class I–deficient F_3_ animals to MCMV infection is not due to a defect in education but to the absence of an activation axis, which is provided by *H2*-D^k^.

### Interplay of inhibitory and activating signals

Activating signals, mediated by the engagement of Ly49P by *H2*-D^k^/*m04*, provided only a marginal enhancement of the NK cell response in the presence of *H2^q^*. Interestingly, we observed a gene dosage effect in the inhibitory action of *H2^q^* that correlated with the level of surface expression of this MHC class I molecule. However, *H2^q^* copy number did not affect the ability of NK cells from *H2-D^k^* transgenic mice (FVB or F3) to eliminate MHC class I deficient target cells; this indicates that *H2^q^* gene dosage does not alter education/licensing of NK cells. By contrast, adoptive transfer experiments demonstrated that *H2^q^* alleles expressed on host cells limit the ability of NK cells to respond to MCMV infection, indicating that the *H2^q^* effect influences NK cell recognition of class I ligands on target cells. This suggests that *H2^q^* inhibitory signals dominate over *H2*-D*^k^*-dependent activating signals emanating from MCMV-infected cells. One possibility is that *H2^q^* inhibitory signals are stronger and/or more frequent than *H2* ^k^-dependent activating signals. Indeed, it has been shown that both the density and the avidity of inhibitory Ly49-ligand pairs determine the strength of inhibition [40]. Alternatively, *H2^q^* MHC class I molecules could compete with H2-D^k^ for binding with the *m04* protein and thus blunt the *m04*/H2-D*^k^*-Ly49P activating axis. We have noted that Ly49P reporter cells are equally stimulated by MCMV-infected MEFs of *H2^k^* or *H2^k^* ^/q^ genotype, which may indicate otherwise (Figure S5). However, these results might not reflect the effect of H2*^q^* molecules on the *H2*-D*^k^*/*m04* complex under physiological conditions. While the molecular details of *H2^q^* inhibition of NK cell function remain unclear, our results suggest a model in which two antagonistic mechanisms are at play in NKC-*H2*-determined resistance to virus infection (Figure 6). One involves enhanced NK cell responses through *H2*-D^k^-mediated activating signals. The other involves dampened NK cell responses through inhibitory Ly49 receptors stimulated by class I *H2^q^* (or *H2^d^*) molecules, which override the effect of the *H2*-D^k^ construct.

### Conclusion

It is puzzling that Ly49 receptors can sense MHC class I molecules on infected cells despite immune-evasion mechanisms elaborated by MCMV that downregulate surface expression of MHC class I molecules. Indeed, mouse strain–specific [41] and cell type–specific [42] differences have been reported in the ability of immunoevasins to inhibit lysis of infected cells by CTLs, indicating that the efficiency of MHC class I downregulation during MCMV infection [43] is context dependent. *In vivo* MCMV replication occurs in a multitude of cell types, and perhaps the ability of the virus to achieve immune avoidance selectively might contribute to the delicate equilibrium of coexistence it has established with the host.

The striking similarities between Ly49 and KIR interactions with their respective MHC-I ligands and how they both affect NK cell function prompted us and other researchers to use the mouse as a model to study NK cell antiviral responses. Our results lend support to clinical and epidemiological studies implicating KIR-HLA interactions of different strengths in determining a hierarchy of NK cell activation with varied effects on the host response against herpersviruses [44], HCV [45], and HIV [46]. Our work also highlights the ability of inhibitory signals to overcome NK cell activation. These regulatory mechanisms would be relevant in conditions where NK cell activation is undesirable during infection or immune disease. For example, activating KIR genotypes have been found to predispose to reactivation of quiescent, opportunistic infections associated with herpesvirus infections in HIV patients [47]), and to fatal outcome following Ebola virus infection [48]; furthermore, they may constitute a risk factor for susceptibility to autoimmunity and certain cancers [49], [50]. Ultimately, our data indicate that, as has been proposed for cancer and autoimmunity, manipulating the balance between inhibitory and activating NK receptor signals represents a possible avenue to harness the therapeutic potential of NK cells against virus infections.

## Materials and Methods

### Ethics statement

The animal protocols and experiments were approved by the Canadian Council on Animal Care (CCAC) and the McGill University Animal Resources Center.

### Animals

MA/My, BALB.K, BALB/c, C57Bl/6 (B6), DBA/J, and AKR mice were purchased from The Jackson Laboratory. FVB/N mice were purchased from Charles Rivers Laboratories. The B6 mice deficient for *H2*-*D^b^K^b^* (B6.*H2* ^0^) were kindly provided by Hidde L. Ploegh (Cambridge, Massachusetts).

### Generation and genetic characterization of congenic mice

BALB-*Cmv3^MA/My^H2^k^* and BALB-*Cmv3^MA/My^H2^d^* were generated by backcrossing the (MA/MyXBALB.K) F1 or (MA/MyXBALB/c) F1 into BALB.K or BALB/c, respectively, for at least ten generations. At each backcross, inheritance of the NKC from parental MA/My mice was genotyped using either the *Ly49e* marker or the *D6mit135* marker [51]. In the progeny, the introgressed portion from parental MA/My mice, which included the NKC, was analyzed using microsatellite markers or by detecting known SNPs. Once the genetic region was reduced from 34 Mb (between *D6MIT36* and *D6MIT59*) to 10 Mb (between *rs13479016* and *rs13479061* SNPs), heterozygous mice were intercrossed to generate the homozygous congenic lines.

### Generation of FVB-Tg(D^k^) transgenic mice and derived F_3_ strains

The *H2*-D^k^ genomic fragment cloned into the PBR22 plasmid was kindly provided by Bernd Arnold (Deutsches Krebsforschungszentrum [DKFZ], Heidelberg, Germany). The 11.5 kb fragment encompassing the D^k^ gene was subsequently purified and injected into fertilized FVB/N mouse eggs. Transgenesis was performed at the Quebec Transgenesis Research Network (QTRN). Transgenic founders were screened by PCR with the primers 5′-cacacgatccagcggctgt-3′ and 5′-ggcccggtctctctctgcag-3′, specific for *H2*-D^k^ exon 3. They were then bred to FVB/N WT mice. To generate F_3_ mice, FVB-Tg(D^k^) and B6.*H2* ^0^ mice were bred to produce F_1_ and F_2_ progeny. To discriminate between the NKC and *H2* regions inherited from the parental strains, the F_2_ mice were genotyped at the NKC region with the *D6Mit61* and *D6Mit52* markers and at the *H2* region with the *D17Mit51* marker; they were also genotyped for the presence or absence of the *H2-D^k^* transgene. Only the mice homozygous for the FVB/N NKC and heterozygous for either the *H2* or the *H2-D^k^* transgene were kept to generate the F_3_ progeny, as listed in Table 1.

### Antibodies and flow cytometry

To prepare splenic leukocytes, spleens were removed aseptically then gently mashed through a 70 µm nylon mesh (BD Bioscience). Red blood cells were lysed with ammonium chloride (Sigma). To isolate lymphocytes from mouse blood, mice were bled from the cheek; blood was collected in RPMI medium containing 15 mM EDTA. Lymphocytes were collected after gradient centrifugation using Histopaque-1077 (Sigma). Fc receptors were blocked with 2.4G2 antibody prior to staining with specific monoclonal antibodies. NK cells were incubated with NKp46 (conjugated to phycoerythrin [PE] or fluorescein isothiocyanate [FITC]) and specific monoclonal antibodies against Ly49A-Biot (YE148), Ly49A/D (12A8), Ly49CIH (14B11), Ly49D (4E5), Ly49G2 (4D11 or AT8), NKG2A/C/E (20d5) or NKG2A/B6 (16A11), CD94 (18D3), or KLRG1 (2F1). NK cells were also incubated with the following isotype control monoclonal antibodies: PE-conjugated golden syrian hamster IgG, FITC- or PE-conjugated mouse IgG2a K, or FITC-conjugated rat IgG2a K (e-Bioscience). *H2*-D^k^ and -D^q^ products were detected by anti-*H2*-D^k^ antibody (15-5-5) from BioLegend and anti-*H2*-D^q^ antibody (KH117) from e-Bioscience. To detect incorporated BrdU on NK cells, mice were scarified 5 or 6 days after MCMV infection; cells were first stained for surface antigens (anti-NKp46 and/or anti-Ly49 receptors) and then fixed, permeabilized, treated with DNase I, and stained with FITC- or allophycocyanin (APC)-conjugated anti-BrdU antibody (clone 3D4; BD Biosciences), according to the manufacturer's protocol. Flow cytometry analysis was performed with a FACSCalibur flow cytometer (BD Biosciences) and data were analyzed using CellQuest (BD Biosciences) or FlowJo (Tree Star). To assess NK cell proliferation *in vivo*, NK cells from the spleen were first enriched by negative selection (Miltenyi Biotec), then incubated with 5 µM CFSE for 15 minutes, washed, and resuspended in PBS. The purity of the NK cells (55%–70%) was evaluated by FACS using anti-NKP46 antibody; 2 million NK cells were then injected intravenously into recipient mice 24 hours before infection with MCMV. The proliferation index, indicating the number of divisions of CFSE-labelled NK cells, was determined using the FlowJo software.

### Viruses and infections

Stock MCMV from mouse salivary glands was prepared by passaging the virus (Smith strain ATCC VR-1399, lot 1698918) twice in BALB/c mice. The virus was prepared from a homogenate of salivary glands 21 days p.i.. Mice aged between 7 and 9 weeks were infected intraperitoneally with 2,000 PFUs of MCMV. The tissue culture-grown viruses [52] *Δm157* MCMV, which lacks the m157 open reading frame (ORF), and *Δm04* MCMV, which lacks the *m04* ORF, have been previously described [23], [53] and were kindly donated by Ulrich H. Koszinowski (Max von Pettenkofer Institute, Munich, Germany) and Stepan Jonjic (Rijeka University, Rijeka, Croatia). Viral titers of the stock virus or mouse organs (spleen and liver) were evaluated *in vitro* by standard plaque assays on a confluent BALB/c MEF monolayer, as previously described [54].

### MEFs: MCMV infection and reporter cell assay

The MEFs used in this work were generated as previously described [52], except for FVB-Tg(D^k^)^+^ transgenic and nontransgenic MEF cells, which were generated from individuals embryos from the progeny of FVB-Tg(D^k^)×FVB wild-type mouse crosses and then genotyped for the presence of the *H2-D^k^* transgene. 2B4 reporter cells expressing Ly49H, Ly49P, Ly49C, or Ly49I were generated as previously described [22], [25], [55]. MEF cultures from AKR, FVB/N *H2*-D^k^ transgenic, FVB/N wild-type, and BALB.K mice were infected with MCMV (Smith strain) or *Δm157* or *Δm04* deletion viruses at a multiplicity of infection (MOI) of 1.0 for 24 hours; they were used to stimulate 2B4 reporter cells as previously described [25]. GFP was detected by flow cytometry and analyzed using the FlowJo software.

### *In vivo* killing of CFSE-labeled MHC class I–deficient cells

Splenocytes from B6.*H2^0^* mice were labeled with 0.4 mM CFSE (CFSE low) in RPMI medium containing 5% FCS; splenocytes from recipient mice were labeled with 4 mM CFSE (CFSE high) in RPMI containing 10% FCS. The splenocytes were then incubated at 37°C for 10 minutes before being washed three times in RPMI containing 10% FCS. Cells (5×10^6^) of each type were mixed, and the mixture (200 µl) was injected intravenously into recipient mice. After 18 hours, spleens were harvested and red blood cells were lysed. The relative percentage of cells in each CFSE population was measured by FACS as previously described [33].

### *In vitro* killing of CFSE-labeled RMA/S cells

NK cells from the spleen were expanded for 5 days in RPMI medium supplemented with 1,000 U/ml human IL-2 (NCI Preclinical Repository). Cells were washed in RPMI and stained with NKp46 antibody to determine the purity of NK cells and to adjust the number of NK cells among strains. RMA/S cells were labeled with 0.4 mM CFSE in RPMI medium containing 5% FCS for 15 minutes at 37°C and washed three times. CFSE-RMA/S and NK cells were cocultured at effector/target ratios of 2∶1, 4∶1, 8∶1, 16∶1, and 32∶1 for 4 hours at 37°C. Specific lysis was determined by the measure of 7-aminoactinomycin D (7-AAD) incorporation (BD) in CFSE-RMA/S cells by flow cytometry, as previously described [56].

### Statistical analysis

For the 137 (FVB/N×BALB/c) F_2_ mice, the contribution of the *NKC* and *H2* loci to the segregation of the phenotype was estimated with the linear model phenotype = *m*+NKC+*H2*+NKC:*H2*+*e*, where NKC and *H2* represent factors that depend on the mode of inheritance proposed, *m* is the common mean value, NKC:*H2* is an interaction term, and *e* is the independent, normally distributed random deviations. For the additive mode of inheritance, the NKC and *H2* represent the number of FVB/N/BALB.K alleles at each locus. For the recessive mode of inheritance, the NKC and *H2* are indicator variables of the homozygous FVB/N and BALB.K backgrounds, respectively. The four possible additive-recessive combinations of *H2*-NKC models, with and without an interaction term, were fitted. We assessed the magnitude of the contribution for each term in the model by its *P* value, obtained by 1 million bootstrapped samples, and partial η^2^. Partial η^2^ was computed as η^2^ = SS_factor_/(SS_factor_+SS_error_), where SS_factor_ is the type 3 associated sum of squares with the factor in the analysis of variance (ANOVA) table, and SS_error_ is the sum of squares corresponding to the residual variation. We carried out statistical and graphical analyses using R software. For other statistical analyses in this work, differences between groups were calculated with two-way ANOVA analysis, followed by Bonferroni after tests. For some of the analyses, unpaired, two-tailed Student's *t*-tests were conducted. Results with a *P* value of <0.05 were considered to be statistically significant.

## Supporting Information

Figure S1 Binding of Ly49-specific monoclonal antibody to MA/My activating receptors. cDNAs encoding MA/My Ly49P, Ly49R and Ly49U receptors were expressed in NFAT-GFP 2B4 T-cell hybridomas [25]. Expression of the three receptors was detected by the anti-Flag M2 monoclonal antibody. Binding of the isotype control monoclonal antibody (red histogram) or the indicated Ly49-specific monoclonal antibody (black histogram) to Ly49P, Ly49R, and Ly49U receptors was assessed by flow cytometry and analyzed using Flowjo software. The percentage of binding is indicated in each histogram.
(1.19 MB EPS)

Figure S2 Frequencies of Ly49+ and KLRG1+NK cells in double congenic mice. Quantification of expression frequency of indicated NK receptors in the parental MA/My strain and BALB-*Cmv3^MA/My^H2^k^* and BALB-*Cmv3^MA/My^H2^d^* congenic mice. Data are presented as mean ± SEM and *P* values of significant differences between groups are indicated.
(0.89 MB EPS)

Figure S3 Lack of NKG2A/C/E and CD94 antibody staining on NK cells from MA/My mice. (A) NKG2A/C/E and CD94 expression on NKp46+ NK cells from MA/My, FVB/N, and DBA/2J (as they carry a NKG2/CD94 deficiency [57]) mice was determined by flow cytometry using the indicated monoclonal antibodies. (B) CD94 and NKG2A RNA expression in enriched NK cells from the indicated mice strains was analyzed by RT-PCR. β-actin was used as an internal control.
(0.87 MB EPS)

Figure S4 Ly49P+2B4 reporter cell stimulation by MCMV-infected MEF cells produced from FVB-Tg(D^k^)^+^ mice. (A) Stimulation of Ly49P reporter cells by co-culture with MEF cells from the indicated backgrounds that were uninfected (black histograms) or MCMV infected at an MOI of 1 for 24 h (grey histograms). Ly49P-specific activation was detected by NFAT-GFP expression using flow cytometry. (B) Stimulation of Ly49P or Ly49H reporter cells by co-culture with FVB-Tg(D*^k^*)*^+^* MEF cells that were uninfected (left) or infected with *Δm157* (middle) or *Δm04* (right) MCMV deletion mutants. Reporter cell stimulation was detected by monitoring expression of GFP by flow cytometry. The percentage of positive cells in each gated population is indicated.
(0.87 MB EPS)

Figure S5 Co-expression of H2-D^q^ and H2-D^k^ in FVB-Tg(D^k^)^+^ mice. Endogenous *H2*-D^q^ (bottom) and transgenic *H2*-D^k^ (top) expression in splenic T and B cells from FVB-Tg(D*^k^*)*^+^* transgenic (black peak) or nontransgenic (black peak) littermates was determined by flow cytometry.
(0.57 MB EPS)

Figure S6 Ly49 receptor expression on NK cells from FVB H2-D^k^ transgenic and nontransgenic mice. (A) The indicated Ly49 specific monoclonal antibodies (black peak) or isotype controls (red peak) were gated on NKp46+ splenic NK cells from FVB-Tg(D*^k^*)^−^ and FVB-Tg(D*^k^*)*^+^* mice and analyzed by FACS. The proportion of Ly49 receptor expression is indicated in each histogram. (B) Quantification of expression frequency of the indicated NK receptors in FVB-Tg(D*^k^*)*^−^* and FVB-Tg(D*^k^*)*^+^* mice.
(1.81 MB EPS)

Figure S7 Ly49 receptor and MHC class I expression on NK cells from F3 mice. The indicated Ly49-specific monoclonal antibodies were gated on NKp46+ splenic NK cells from F3 mice and the proportion of expression is indicated in each histogram. Right panels: expression of MHC-I *H2*-D^q^ and *H2*-D^k^ molecules on total splenocytes was determined. 2–3 mice per genotype were analyzed. We found that the expression of the activating and the inhibitory receptors were almost comparable between strains for the exception of Ly49G which was barely detectable in the *Cmv3^FVB^/H2^0^*/Tg(D*^k^*)*^−^* mice using both anti- Ly49G antibodies (LGL-1 and AT8 (data not shown)). This receptor is perfectly expressed in the B6.*H2^0^* parental strain (data not shown) and doesn't seem to be correlated with a defect of NK maturation since the Killer cell lectin-like receptor G1 (KLRG1) is equally expressed between the *Cmv3^FVB^*/*H2^0^*/Tg(D*^k^*)*^+^* and the *Cmv3^FVB^*/*H2^0^*/Tg(D*^k^*)*^−^* mice.
(1.37 MB EPS)

Figure S8 NK cell–dependent MCMV infection control in congenic and F3 mice. (A) Viral loads in spleens (top) and livers (bottom) 3 d post-infection in MCMV-resistant MA/My progenitor, BALB-*Cmv3^MA/My^H2^k^* congenic, and *Cmv3^FVB^/H2^0^*/Tg(D*^k^*)*^+^* transgenic mice that were NK cell depleted (white squares) or not (black circles) with anti-asialo GM1 antibody. (B) Number of NK cells per spleen (top) and BrdU incorporation (bottom) at 7 d post-MCMV infection in MCMV-resistant mice of the indicated genotypes. For the number of NK cells, data are presented as mean ± SEM and statistically significant differences between groups are indicated. For BrdU incorporation data are represented by fold increase between noninfected and infected animals. Results shown are representative of 1–2 experiments.
(1.56 MB EPS)

Figure S9 Effect of Ly49-specifc monoclonal antibody depletion in MA/My mice during the course of MCMV infection. MCMV viral load was assessed in untreated MA/My mice (mock) or treated with the indicated monoclonal antibody prior to MCMV infection. Viral load in spleen was determined by plaque assay after 3 d of infection.
(1.47 MB EPS)
